# Efficacy of High-Intensity Laser Therapy Combined With Plantar Fascia Stretching Exercises in the Treatment of Plantar Fasciitis: Randomized, Double-Blind, Sham-Controlled Trial

**DOI:** 10.2196/77419

**Published:** 2026-02-20

**Authors:** Nantaporn Jitpimolmard, Phonlawat Ouemphancharoen, Preeda Arayawichanon

**Affiliations:** 1 Department of Rehabilitation Medicine, Faculty of Medicine, Khon Kaen University, 123 Mittraphap Road, Mueang, Khon Kaen, 40002, Thailand, 66 43366123

**Keywords:** plantar fasciitis, high-intensity laser therapy, lasers, pain measurement, ultrasonography, fascia, foot, ankle

## Abstract

**Background:**

Plantar fasciitis causes heel pain and functional limitations; conservative treatment typically includes plantar fascia and calf stretching. High-intensity laser therapy (HILT) offers deeper photobiomodulation and potential tissue-healing benefits, but robust evidence of added clinical benefit remains limited.

**Objective:**

This study aims to evaluate the efficacy of HILT combined with plantar fascia stretching exercises compared with a sham control for the treatment of plantar fasciitis.

**Methods:**

This study was designed as a randomized, double-blind, sham-controlled trial conducted at the outpatient clinic of a university hospital. Participants were randomly allocated into 2 groups: the HILT group and the sham treatment group. Both groups received 9 treatment sessions over 3 weeks. The HILT group received active laser therapy, while the sham group received identical treatment without laser emission. The HILT used a wavelength of 1064 nm in continuous mode, with a power output of 12 W applied for 250 seconds, delivering an energy density of 120 J/cm² applied to a 25-cm² area for a total energy of 3000 J. In addition to the assigned interventions, all participants performed a standardized self-stretching exercise program targeting the plantar fascia and Achilles tendon throughout the study period. The primary outcome was pain intensity measured using a visual analog scale. Secondary outcomes included ultrasonographic measurement of plantar fascia thickness (PFT) and the subjective foot and ankle ability measure (FAAM) score, recorded before and immediately after the intervention.

**Results:**

A total of 34 patients diagnosed with unilateral plantar fasciitis were enrolled in this study. Based on intragroup comparison, both groups demonstrated statistically significant improvements in all outcomes compared with baseline (*P*<.001). However, no significant differences were found between the 2 groups across all outcomes. The mean difference in pain reduction, measured by the visual analog scale, was –35.3 (95% CI –45.3 to –25.0) mm in the HILT group and –30.4 (95% CI –46.3 to –14.4) mm in the sham group (–5.0 mm, 95% CI –14.3 to 4.3; *P*=.59). Similarly, reductions in PFT and improvements in FAAM scores showed no significant differences between groups (mean difference –0.02 mm, 95% CI –0.2 to 0.1; *P*=.90 and 5.6 points, 95% CI –1.1 to 12.4; *P*=.40, respectively).

**Conclusions:**

There was no additional clinical effectiveness of HILT on pain reduction, decreased PFT, or increased FAAM scores compared with sham laser when combined with standard stretching exercises for plantar fasciitis and the Achilles tendon.

## Introduction

Plantar fasciitis is a common and often disabling condition characterized by pain at the plantar aspect of the heel, which can substantially limit daily activities [1]. Risk factors include increasing age, elevated body mass index, prolonged standing, and foot posture abnormalities that increase stress on the plantar fascia [2]. First-line management is conservative and heterogeneous, encompassing manual therapy, night splints, taping, orthoses, and exercise programs; among these, stretching of the plantar fascia and associated calf musculature is widely recommended and frequently used in clinical practice [3].

For this study, passive stretching was chosen as the standardized backbone intervention. Mechanistically, stretching directly targets gastrocnemius–soleus tightness and plantar fascia strain, two important contributors to symptom persistence, and has been shown in clinical studies to reduce pain and improve function in the short to medium term [4]. Practically, passive stretching is easily standardized, low cost, self-administered, well tolerated, and associated with good patient adherence compared with more resource-intensive or operator-dependent modalities (eg, manual therapy or supervised exercise programs). Using a single, well-established conservative treatment as the common background therapy therefore reduces between-participant variability and more clearly isolates any incremental effect of the adjunctive intervention under study.

Adjunctive physical modalities—including extracorporeal shockwave therapy, ultrasound, and low-intensity laser therapy—are commonly used to enhance recovery [5]. Laser therapy delivers photons that may stimulate cellular processes involved in tissue repair, including increased adenosine triphosphate production, enhanced collagen synthesis, and improved microcirculation [6-8]. Systematic reviews indicate that low-intensity laser therapy can reduce pain in plantar fasciitis [5,9-12]. In theory, high-intensity laser therapy (HILT) has greater tissue penetration and may produce stronger collagen stimulation, greater blood flow effects, and more pronounced anti-inflammatory responses than low-intensity devices [13,14]. However, studies comparing HILT with low-intensity laser or sham treatment have reported conflicting results [15,16], and recent meta-analyses have not comprehensively evaluated HILT for plantar fasciitis.

Prior HILT studies have often been limited by methodological weaknesses, inconsistent control treatments, and a scarcity of objective outcomes such as ultrasound-measured plantar fascia thickness (PFT) or functionally relevant measures. To address these gaps, this randomized, sham-controlled trial evaluated the efficacy of HILT added to a standardized passive stretching program compared with sham laser plus the same conservative regimen. We hypothesized that adjunctive HILT would produce greater improvements in pain, functional capacity, and PFT compared with conservative care using sham laser.

## Methods

### Study Design

This study was a prospective, randomized, double-blinded (participant and assessor), sham-controlled trial conducted in an outpatient rehabilitation setting at Srinagarind Hospital, Khon Kaen University.

### Ethical Considerations

The study protocol was approved by the Khon Kaen University Ethics Committee (HE621318). Written informed consent was obtained from all participants prior to enrollment. Participant privacy and confidentiality were strictly maintained. All data were deidentified prior to analysis, and personal identifiers were removed. The dataset was stored securely and was accessible only to authorized members of the research team. Participants received compensation of ฿900 for their participation. Based on an exchange rate of US $1 to ฿31, this is approximately US $29 per participant. Compensation was provided regardless of study completion.

The study adhered to the CONSORT (Consolidated Standards of Reporting Trials) guidelines.

### Participants

A total of 36 participants diagnosed with unilateral plantar fasciitis were recruited between January 2020 and September 2020.

#### Inclusion Criteria

Participants were eligible for inclusion if they met all of the following criteria:

- Adults aged 18 years or older
- Foot pain persisting for a minimum of 6 weeks
- Diagnosis of plantar fasciitis confirmed by a rehabilitation medicine physician based on clinical history, characterized by pain primarily felt during the first steps after waking, which worsens with prolonged weight-bearing activities throughout the day [17]
- Tenderness at the medial calcaneal tubercle upon palpation [17]

#### Exclusion Criteria

Participants were excluded from the study if they met any of the following criteria:

- History of foot surgery
- Chronic inflammatory arthritis (eg, rheumatoid arthritis, gout, systemic lupus erythematosus)
- Conditions affecting sensory perception (eg, diabetes mellitus with peripheral neuropathy)
- History of corticosteroid injection to the plantar fascia within the preceding 6 months
- Neuropathic pain in the foot, or evidence of intrinsic foot muscle weakness
- Pregnancy

### Baseline Assessment

At baseline, the following data were collected: age, gender, body mass index, side with the most severe pain, and Foot Posture Index (FPI-6) score.

### Randomization and Blinding

Participants were randomly assigned to either the HILT group or the sham laser (control) group using a permuted block randomization method with a block size of 4. The randomization sequence was computer generated and concealed within sequentially numbered, opaque, sealed envelopes. Envelopes were opened by the treating therapist immediately prior to the first treatment session. Participants were blinded to their treatment allocation. The assessor performing outcome measurements was also blinded to treatment allocation.

### Intervention

#### Overview

All participants received 9 treatment sessions administered every 3 days. Both groups received a standardized home exercise program consisting of plantar fascia and Achilles tendon stretching exercises. Participants were instructed to perform the following stretch while seated or supine: with a towel or strap around the forefoot, the knee was extended and the forefoot pulled toward the body, maintaining dorsiflexion for 30 seconds. The exercise was performed for 10 repetitions per session, once daily. Adherence to the home exercise program was monitored using patient logs.

#### HILT Group

HILT was administered by a trained medical doctor using the BTL-6000 high-intensity laser device (BTL Industries) operating at a wavelength of 1064 nm. The treatment parameters were as follows: continuous mode, power output of 12 W applied for 250 seconds, energy density of 120 J/cm² delivered over a 25-cm² area, resulting in a total energy of 3000 J [15,16]. The participant was positioned prone with the foot in a neutral position. The laser was applied using a 10-mm pen applicator and swept over the calcaneal insertion and along the medial border of the plantar fascia.

#### Sham Laser Group

The sham treatment mimicked the HILT procedure, including the visual and auditory cues of the laser device, but without actual laser energy delivery. The same treatment protocol as the HILT group was followed in terms of positioning, applicator movement, and device sounds.

#### Medication and High Plantar Fascial Loading Activity

Participants were instructed to maintain their usual pain medication regimen, which was limited to 500 mg of paracetamol, and not to exceed 3000 mg per day. They were also advised to strictly refrain from strenuous exercises that involve high plantar fascial loading. Crucially, the use of anti-inflammatory drugs was strictly prohibited during the study per the protocol. The number of pain medication pills taken was recorded throughout the study period.

### Outcome Measures

Outcomes were assessed at baseline and immediately postintervention by a blinded assessor. Pain intensity was measured using a 100-mm visual analog scale (VAS). Participants were asked to recall the pain experienced during the first 3 steps taken in the morning. Scores were assessed immediately preintervention and again via telephone 1 day after the final session to capture pain during participants’ first steps in the morning following the treatment period.

PFT was measured by ultrasound using a LOGIQ E Premium system (GE Healthcare) with a 5 MHz linear probe at the calcaneal insertion. Participants were positioned prone with feet in a neutral position, and the feet were placed at the end of the examination bed. The ultrasound probe was placed perpendicular to the plantar fascia on the plantar surface, and the thickness of the thickest portion was measured [18]. The reported value is the mean of 3 measurements.

Foot and ankle function was assessed using the activities of daily living subscale of the Thai version of the foot and ankle ability measure (FAAM). The FAAM subjective form evaluates foot and ankle function for daily activities and sports; however, only the activities of daily living subscale was used in this study. The reliability of the FAAM was examined by Arunakul et al [19], who reported strong internal consistency.

### Data Analysis

All analyses were conducted using Python (version 3.10), executed in Google Colaboratory. Continuous variables are presented as mean (SD) or as median (IQR) for nonnormally distributed data, while categorical variables are presented as frequency (percentage). Normality of continuous data was assessed using the Shapiro-Wilk test.

The primary analysis followed the intention-to-treat (ITT) principle. Missing outcome data (5%) were addressed using multiple imputation by chained equations with 20 imputations. The imputation model included baseline outcome values, treatment group, and relevant covariates. Each imputed dataset was analyzed separately, and the results were pooled using Rubin’s rules.

Between-group comparisons of continuous posttreatment outcomes (primary and secondary) were conducted using independent *t* tests when distributional assumptions were met. For nonnormally distributed outcomes, between-group comparisons used the Mann-Whitney *U* test on observed data, with additional sensitivity analyses performed on the imputed datasets. Within-group changes were evaluated using paired *t* tests or the Wilcoxon signed rank test, as appropriate.

A per-protocol analysis including participants who completed at least 7 treatment sessions was conducted as a sensitivity analysis. Effect sizes (Cohen *d*) and 95% CIs were reported for parametric comparisons. All statistical tests were 2-tailed, and a *P* value <.05 was considered statistically significant.

### Sample Size Calculation

Based on prior studies [15], a sample size of 16 participants per group was required to detect a mean difference of 15 (SD 15) mm on the VAS, with 80% power and a 2-sided α of .05. To allow for an anticipated 10% dropout rate, 36 participants (18 per group) were recruited.

### Withdrawal and Discontinuation Criteria

Participants could withdraw from the study at any time without affecting their care. Discontinuation criteria included withdrawal of consent or adverse effects (eg, burns, eye irritation).

## Results

**Figure 1.**
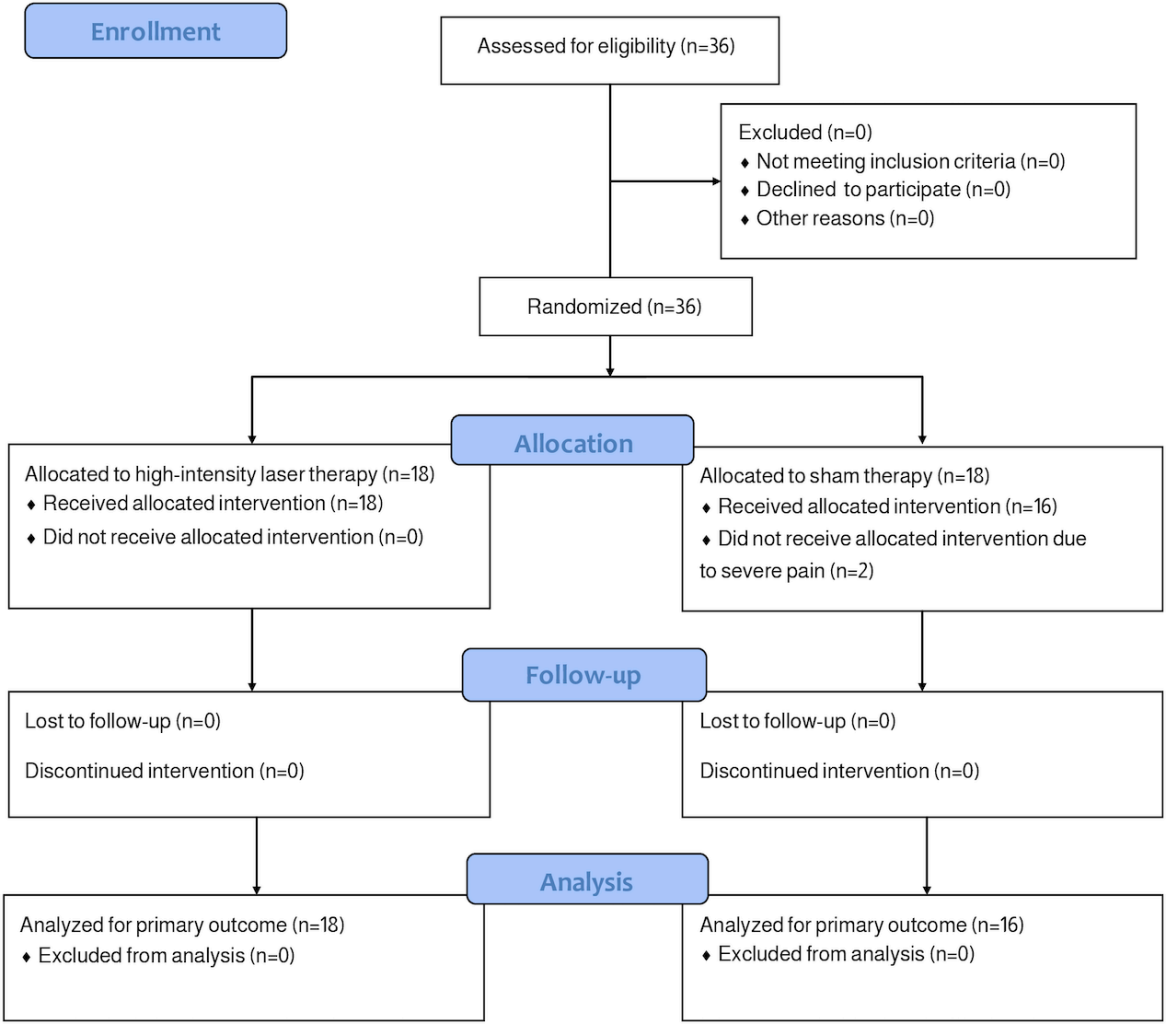
CONSORT (Consolidated Standards of Reporting Trials) diagram outlining the identification, enrollment, and allocation of participants to the 2 groups: high-intensity laser therapy group and sham group.

A total of 36 participants were enrolled, with 34 participants completing the study. Two participants withdrew due to severe pain and opted for alternative treatments (Figure 1).

The HILT group included 18 participants (n=2 men, n=16 women; mean age 46, SD 11.4 years), while the sham laser group included 16 participants (n=1 man, n=15 women; mean age 49, SD 13.0 years). The mean body mass index was 23.3 (SD 2.9) kg/m² in the HILT group and 26.0 (SD 2.8) kg/m² in the sham laser group (*P*=.20). The FPI-6 mean scores were –0.4 (SD 1.1) in the HILT group and –0.6 (SD 1.1) in the sham group, indicating a slightly supinated foot posture [20]. Moreover, no patients in our study had markedly supinated or pronated feet. There was no significant difference in the duration of foot pain, with a mean of 14.7 (SD 4.2) weeks in the HILT group and 14.8 (SD 4.4) weeks in the sham group (Table 1).

**Table 1.** Demographic characteristics of the 2 study groups.

Characteristics	High-intensity laser therapy group (n=18)	Sham group (n=16)	*P* value
Age (y), mean (SD)	46 (11.4)	49 (13.0)	.52
Gender, n (%)			.61
Male	2 (11.1)	1 (6.3)	
Female	16 (88.9)	15 (93.7)	
FPI-6^a^ (points), mean (SD)	–0.4 (1.1)	–0.6 (1.1)	.51
BMI (kg/m²), mean (SD)	23.3 (2.9)	26.0 (2.8)	.21
Duration of foot pain (wk), mean (SD)	14.7 (4.2)	14.8 (4.4)	>.99

a FPI-6: Foot Posture Index.

The ITT analysis (n=36; pooled across 20 imputations) showed the following posttreatment results. For pain (VAS), the mean posttreatment score was 23.0 (SD 17.5) mm in the HILT group versus 26.8 (SD 20.7) mm in the sham group. The between-group mean difference (laser and sham groups) in posttreatment VAS was –5.0 mm (95% CI –14.3 to 4.3; *P*=.59). Cohen *d* for the between-group difference at posttreatment (pooled SD) was small (d≈–0.20).

Mean PFT decreased from 4.8 (SD 1.4) mm to 4.1 (SD 1.2) mm (mean change –0.7 mm, 95% CI –1.0 to −0.5; *P*<.001) in the HILT group. For PFT, the median decreased from 5.3 (IQR 4.5‐5.4) mm to 4.5 (IQR 3.7‐4.7) mm (median change –0.7 mm, 95% CI –1.0 to −0.5; *P*<.001) in the sham group. The between-group difference in change was –0.02 mm (95% CI –0.2 to 0.1; *P*=.90). Because values in the sham group were nonnormally distributed, a nonparametric test (Mann-Whitney *U* test) was used for between-group comparisons of this outcome, and Cohen *d* was not reported for this comparison.

For FAAM, the mean posttreatment scores were 82.3 (SD 12.8) in the HILT group and 82.8 (SD 17.7) in the sham group. Within-group improvements were significant in both groups. The mean change was 18.7 (95% CI 10.4 to 27.1; *P*<.001) in the HILT group and 14.5 (95% CI 3.2 to 25.8; *P*=.02) in the sham group. The between-group mean difference in change was 5.6 (95% CI –1.1 to 12.4; *P*=.40). Cohen *d* for the between-group posttreatment comparison was very small (d≈0.03; Table 2).

**Table 2.** Comparison of pain intensity measured by the 100-mm VAS, plantar fascial thickness, and FAAM score.

Outcomes	High-intensity laser therapy group (n=18)				Sham group (n=16)				High-intensity laser therapy versus sham	
	Before, mean (SD)	After, mean (SD)	Mean difference (95% CI)	*P* value	Before, mean (SD)	After, mean (SD)	Mean difference (95% CI)	*P* value	Between-group mean difference (95% CI)	*P* value
VAS^a^ (mm)	58.3 (19.1)	23.0 (17.5)	–35.3^b^ (–45.3 to –25.0)	<.001	57.1 (22.2)	26.8 (20.7)	–30.4^b^ (–46.3 to –14.4)	.001	–5.0 (–14.3 to 4.3)^c^	.59
Plantar fascia thickness (mm)	4.8 (1.4)	4.1 (1.2)	–0.7^b^ (–1.0 to –0.5)	<.001	5.3 (4.5 to 5.4)^d^	4.5 (3.7 to 4.7)^d^	–0.7^e^ (–1.0 to –0.5)	<.001	–0.02 (–0.2 to 0.1)^c^	.90
FAAM^f^ (point)	63.6 (13.6)	82.3 (12.8)	18.7^b^ (10.4 to 27.1)	<.001	68.3 (23.8)	82.8 (17.7)	14.5^b^ (3.2 to 25.8)	.02	5.62 (–1.1 to 12.4)^c^	.40

a VAS: visual analog scale.

b Paired *t* test was used.

c Independent *t* test was used.

d Median (IQR).

e Wilcoxon signed rank test was used.

f FAAM: foot and ankle ability measure.

The per-protocol analysis (n=34 completers) produced results consistent with the ITT analysis; between-group differences in change scores remained small and nonsignificant for VAS, PFT, and FAAM, supporting the robustness of the findings. Among participants with plantar fasciitis who had a history of strenuous weight-bearing exercise, 1 participant (a runner) in the HILT group was advised to refrain from such activity during the treatment period. Furthermore, 2 participants (1 from each group) reported taking paracetamol at a dosage of 2000 mg per day for 2 days. No adverse events (eg, increased pain, skin burns) were reported in the HILT group. Two participants in the sham group withdrew owing to pain intolerance. Adherence to the exercise protocol did not differ significantly between groups: there were a mean 15.1 (SD 3.8) sessions in the HILT group versus 17.5 (SD 3.7) in the sham group (*P*=.06).

## Discussion

This randomized controlled trial assessed the effectiveness of HILT combined with conventional therapy (stretching exercises) compared with sham laser plus conventional therapy in patients with plantar fasciitis. A key strength of our methodology, which distinguishes it from many prior trials, was the inclusion of objective structural outcomes (ultrasound) and validated quality-of-life assessment (FAAM), coupled with a rigorous sham control. Both treatment arms showed significant improvements in pain scores, a reduction in PFT, and an increase in foot and ankle function following treatment. However, HILT did not demonstrate superiority over the sham intervention.

This primary finding—that HILT offered no clear added benefit—aligns with several previous investigations. For instance, Tkocz et al [21], studying chronic heel pain (including plantar fasciitis and calcaneal spurs) using a HILT device at a lower power (7 W) and high total energy, similarly found no clear advantage of HILT over sham combined with ultrasound. Furthermore, Naruseviciute and Kubilius [16] also reported no superiority of HILT versus low-intensity laser therapy regarding either pain or fascial thickness. Conversely, Akkurt et al [22] observed improved outcomes when HILT was combined with insoles; however, the absence of a true sham laser control in that study makes it impossible to definitively exclude a significant placebo contribution.

The reasons for this consistent lack of incremental benefit are likely multifactorial. High-intensity laser protocols vary widely across trials concerning power, energy per session, mode, number of sessions, and application technique [15,16,21,22]. This substantial heterogeneity may partly explain discrepant results in the literature, as these parameters dictate whether the tissue effects are primarily photobiomodulatory or thermal. Nevertheless, the majority of trials to date do not show a consistent, clinically meaningful incremental effect of HILT over standard conservative care. Future research must prioritize robust dose-finding studies, provide complete dosimetry and application details, and include mechanistic measures to determine whether any specific parameter set reliably improves outcomes in plantar fasciitis before routine adoption.

Beyond methodological variations, anatomical considerations may temper the potential benefit of HILT. The plantar fascia lies only a few millimeters below the skin, varying by location and individual subcutaneous thickness. Consequently, the deeper penetration advantage often ascribed to HILT may be less relevant for plantar fasciitis compared with deeper targets (eg, knee osteoarthritis), where reaching intra-articular structures is more pertinent [23]. This superficial anatomical location of the target may inherently limit the potential incremental benefit of HILT in this disease.

Given that all 3 primary outcomes improved significantly in both study arms, it strongly suggests that the standardized stretching program was the major driver of clinical benefit. Plantar fascia and calf stretching target the gastrocnemius–soleus complex and the fascia because tightness increases tensile strain on the arch [4]. Mechanistically, regular stretching likely contributed to the reduction in pain and functional gains by increasing ankle dorsiflexion, lowering passive tension transmitted to the fascial insertion, and improving tissue viscoelasticity.

The consistent improvements observed in our objective and patient-centric measures validate the effectiveness of this conservative approach. The reduction in PFT, quantified objectively via ultrasound, confirms that the therapy successfully induced a positive structural change in the affected tissue, moving beyond mere symptomatic relief. PFT is considered the anatomical hallmark of chronic plantar fasciitis (reflecting chronic microtearing and disorganized collagen repair) [18]. Similarly, the significant increase in scores on the FAAM further substantiates the clinical relevance of the treatment. Since the FAAM is a validated patient-reported outcome measure focusing specifically on function and participation in daily living and sports activities [19], the documented improvements indicate that the structural and pain changes successfully translated directly into better functional capacity for participants.

An alternative explanation for the null between-group difference lies in statistics. The study was powered to detect a 15-mm VAS difference, yet the observed between-group difference was statistically small (only –5.0 mm). This small effect size combined with substantial variability reduced the power to find a statistically significant difference. Additionally, because many participants improved substantially with stretching (and some used short courses of acetaminophen), a ceiling effect is possible, leaving little room for additional measurable improvement attributable solely to HILT. Reporting responder rates, change-score distributions, and conducting larger trials or trials enrolling participants with higher baseline pain would help determine whether a clinically meaningful subgroup benefit exists.

Most participants in our sample had near-normal alignment with slight supination, and baseline alignment was balanced between the 2 groups, so imbalance in foot posture is unlikely to account for the null between-group findings. Nonetheless, because plantar fascia loading differs across foot types and many patients with plantar fasciopathy present with pes planus or overpronation [2], the sample’s alignment profile may limit external validity. Future trials should objectively quantify foot posture (eg, FPI-6, navicular drop, calcaneal eversion, or dynamic pressure measures), consider stratified randomization or prespecified subgroup analyses by alignment, and, where appropriate, tailor background conservative care to alignment to test whether foot posture modifies the effect of adjunctive treatments such as HILT.

In terms of safety and adherence, a small number of participants used brief acetaminophen therapy, and 1 HILT participant was advised to avoid strenuous running during treatment. This activity modification is an independent potential confounder. Importantly, however, no adverse events were reported among HILT recipients, supporting short-term tolerability in our sample. Although 2 participants in the sham group withdrew because of pain intolerance, both ITT and per-protocol sensitivity analyses produced similar results, supporting the robustness of our conclusions.

Finally, this study assessed outcomes only immediately postintervention, meaning the durability of effects remains unknown. Furthermore, the lack of standardized HILT protocols for plantar fasciitis complicates cross-trial comparison. Future research must prioritize longer follow-up, dose-finding studies, standardized reporting of dosimetry, and adequately powered trials (including stratified or targeted enrollment) to identify the specific patient subgroups most likely to benefit from HILT.

This study provides high-quality evidence that HILT does not offer additional benefits over standard stretching exercises in the treatment of plantar fasciitis. Given the statistically similar outcomes between the HILT and sham laser groups, our findings reinforce the effectiveness of conservative treatment approaches as the primary management strategy for plantar fasciitis.

## Supplementary material

10.2196/77419 Checklist 1 CONSORT-EHEALTH (V 1.6.1) checklist.

## Abbreviations

FAAM: foot and ankle ability measure
FPI-6: Foot Posture Index
HILT: high-intensity laser therapy
ITT: intention-to-treat
PFT: plantar fascia thickness
VAS: visual analog scale
